# Shared inflammatory architecture and therapeutic tensions between psoriasis and Crohn’s disease

**DOI:** 10.3389/fimmu.2026.1871406

**Published:** 2026-06-26

**Authors:** Xiaoou Wang, Jitong Li, Keying Yu, Xianbo Wu, Quan Luo

**Affiliations:** 1 Yanbian Hospital of Traditional Chinese Medicine, Yanji City Hospital of Traditional Chinese Medicine, Yanji, Jilin, China; 2 The Affiliated Hospital of Changchun University of Chinese Medicine, Changchun, Jilin, China; 3 Chengdu Sport University, School of Sports Medicine and Health, Chengdu, Sichuan, China; 4 West China School of Medicine, Sichuan University, Sichuan University Affiliated Chengdu Second People’s Hospital, Chengdu Second People’s Hospital, Chengdu, Sichuan, China

**Keywords:** Crohn’s disease, cross-organ inflammation, gut-skin axis, immune-mediated inflammatory diseases, psoriasis, shared immune pathways

## Abstract

Psoriasis and Crohn’s disease are chronic immune-mediated inflammatory diseases affecting distinct barrier organs, yet epidemiological, genetic, transcriptomic, and therapeutic evidence supports partial immune convergence between them. This review argues that the relationship between psoriasis and Crohn’s disease reflects partial immune convergence shaped by tissue context, rather than a single shared disease entity. TNF-α and IL-23-centered type 17 immunity represent the most clinically relevant shared upstream programs, whereas downstream effector pathways, especially IL-17-related responses, are shaped differently by skin and gut barrier architecture, resident immune ecology, microbial exposure, and repair demands. We discuss the gut–skin axis with caution: barrier dysfunction, dysbiosis, microbial metabolites, and immune-cell trafficking may connect skin and intestinal inflammation, but direct causal evidence in humans remains limited. TNF inhibitors, IL-12/23 blockade, and selective IL-23 inhibitors are the most plausible options for selected patients requiring treatment compatible with both skin and gut disease, whereas IL-17 blockade and paradoxical psoriasiform reactions illustrate organ-specific therapeutic tensions. Future progress will depend on patient stratification using clinical phenotypes, biomarkers, tissue profiling, and treatment history to identify patients in whom skin and intestinal inflammation are driven by overlapping immune mechanisms.

## 1 Introduction

Although psoriasis and Crohn’s disease (CD) primarily affect the skin and the gastrointestinal tract, respectively, neither is now regarded as a purely organ-confined inflammatory disorder. Psoriasis is a chronic immune-mediated inflammatory skin disease whose clinical impact extends far beyond the skin, and it is frequently accompanied by systemic comorbidities, including psoriatic arthritis, cardiometabolic abnormalities, and substantial psychosocial burden (1). In this review, psoriasis refers primarily to cutaneous psoriasis unless psoriatic arthritis is explicitly specified. Psoriatic arthritis is discussed separately when joint involvement affects systemic inflammatory burden, comorbidity assessment, or therapeutic selection. CD, one of the major subtypes of inflammatory bowel disease (IBD), is characterized by chronic relapsing mucosal inflammation, cumulative disease burden over time, and marked adverse effects on quality of life and healthcare utilization (2, 3). More importantly, both conditions are now recognized as part of the broader spectrum of immune-mediated inflammatory diseases (IMIDs) (3, 4). This conceptual framework suggests that the relationship between psoriasis and CD should not be interpreted solely on the basis of the organs involved but rather examined within the broader context of systemic immune dysregulation and cross-organ inflammatory networks.

Epidemiological and clinical evidence indicates that the association between psoriasis and CD is unlikely to represent a simple coincidence. Population-based studies have shown an increased prevalence of IBD among patients with psoriasis, with stronger associations observed in those with more severe skin disease (5). Conversely, patients with Crohn’s disease may develop cutaneous manifestations, including inflammatory bowel disease-associated dermatoses and psoriasis-like lesions, during the disease course or in the context of biologic therapy (6). These observations support partial immune convergence between psoriasis and Crohn’s disease, but they do not establish a single shared disease process.

**Figure 1:**
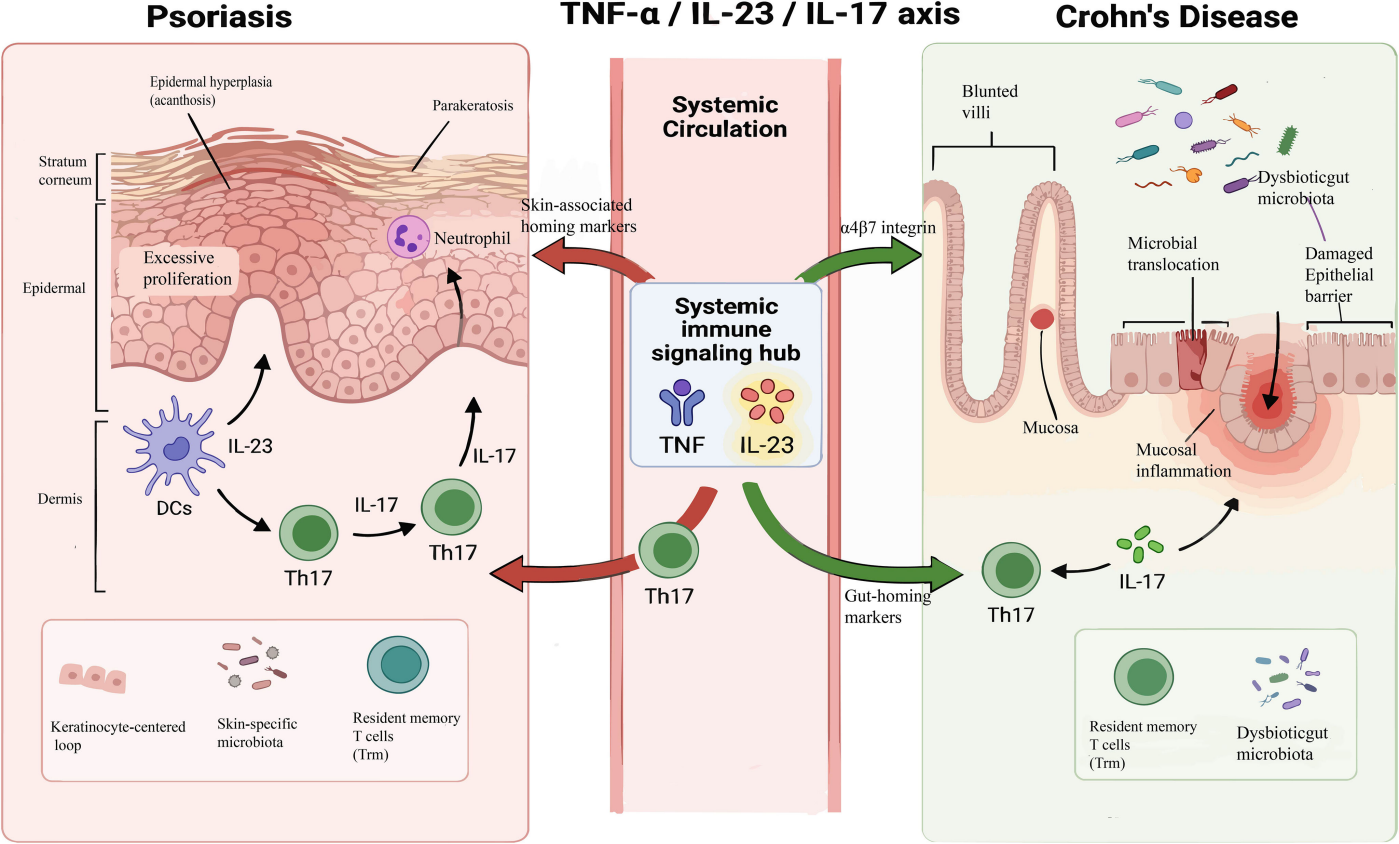
Shared immune axis between cutaneous psoriasis and Crohn’s disease and tissue-specific differentiation. The schematic summarizes how systemic TNF-α/IL-23/IL-17 signaling may connect psoriatic skin inflammation and Crohn’s disease while still producing organ-specific outcomes. In psoriasis, dendritic cell-derived IL-23 sustains Th17 responses and IL-17-mediated keratinocyte activation, contributing to epidermal hyperplasia, parakeratosis, neutrophil recruitment, and local inflammatory amplification. In Crohn’s disease, related type 17 immune signals interact with dysbiotic gut microbiota, mucosal barrier injury, microbial translocation, and mucosal inflammation. The central circulation indicates shared inflammatory communication, whereas tissue-specific homing markers and resident immune contexts explain why similar upstream signals are filtered differently in the skin and gut. TNF-α, tumor necrosis factor α; IL, interleukin; DCs, dendritic cells; Th17, T helper 17 cells; CCR, C-C chemokine receptor; α4β7, α4β7 integrin; TRM, tissue-resident memory T cells.

Rather than listing every mediator reported in both diseases, this review focuses on the evidence that is most relevant to mechanism and treatment. We distinguish pathways with clear cross-organ relevance from associations that remain indirect, context-dependent, or insufficient for therapeutic extrapolation. We first synthesize epidemiological, clinical, genetic, transcriptomic, and spatial evidence for cross-organ immune convergence. We then analyze TNF-α, IL-23-centered type 17 immunity, barrier dysfunction, microbiota-related signals, and tissue-specific inflammatory niches. Finally, we discuss how these mechanisms inform patient stratification and therapeutic selection, with particular attention to IL-17-related intestinal concerns, paradoxical psoriasiform reactions, and other organ-specific therapeutic tensions. We argue that psoriasis and Crohn’s disease share several upstream inflammatory pathways, particularly TNF-α and IL-23/type 17 immunity. Their clinical meaning, however, depends on how these pathways operate within keratinocyte-dominated skin inflammation or within intestinal mucosal injury, microbial exposure, and repair. These relationships are summarized in **Figure 1**.

## 2 Evidence supporting the association between psoriasis and Crohn’s disease beyond simple comorbidity

### 2.1 Epidemiological and clinical evidence for cross-organ association

Epidemiological evidence supports a bidirectional association between cutaneous psoriasis and Crohn’s disease. From the psoriasis side, population-based data show that Crohn’s disease is more common in patients with psoriasis, with a stronger association in moderate-to-severe cutaneous psoriasis than in mild disease (5). From the Crohn’s disease side, a matched cohort study reported a substantially higher adjusted risk of new-onset cutaneous psoriasis in patients with Crohn’s disease than in non-IBD controls, and this association persisted after excluding patients exposed to TNF inhibitors (7). Clinical evidence further suggests that Crohn’s disease with concomitant cutaneous psoriasis may identify a subgroup with more extensive intestinal involvement, perianal disease, higher inflammatory burden, metabolic abnormalities, and poorer response to infliximab (8). However, these data remain observational and do not establish a fixed causal direction or a distinct “Crohn’s disease with psoriasis” subtype. Thus, epidemiological and clinical data support a non-random association between cutaneous psoriasis and Crohn’s disease, particularly in patients with greater inflammatory burden. However, these studies do not establish whether one disease drives the other, whether both arise from shared predisposition, or whether part of the association reflects treatment exposure and surveillance bias.

### 2.2 Genetic, transcriptomic, and spatial evidence supporting immune convergence

Genetic evidence supports pathway-level convergence between psoriasis and Crohn’s disease. A cross-trait GWAS and pleiotropy analysis identified shared genetic signals between psoriasis and immune-mediated gastrointestinal diseases, with candidate genes enriched in immune-related tissues and cell types (9). Disease-specific GWAS studies also show that susceptibility to psoriasis and inflammatory bowel disease is concentrated in immune-regulatory pathways, including cytokine signaling, lymphocyte differentiation, and inflammatory regulation (10, 11). In particular, IL-23-related biology provides a plausible shared upstream module, as IL23R and related pathway components have been implicated in both psoriasis and inflammatory bowel disease susceptibility (12). These findings support immune convergence at the pathway level rather than a single shared genetic cause.

Transcriptomic and integrative bioinformatic studies further support this interpretation. Shared gene-signature analyses have identified overlapping inflammatory programs between psoriasis and Crohn’s disease, particularly those related to immune activation and IL-17/IL-23-associated pathways (13). Integrated biomarker and machine-learning analyses have also suggested that the two diseases may share selected molecular markers and immune-cell infiltration patterns (14). However, these transcriptomic overlaps should not be interpreted as proof of identical disease biology. They indicate programmatic convergence, but they do not explain how similar upstream immune signals generate different tissue phenotypes.

Spatial transcriptomic studies add a tissue-level dimension to this question. In psoriasis, spatially resolved analyses show that inflammatory programs are not uniformly distributed across lesional skin, but are organized within epidermal–dermal immune niches involving keratinocytes, stromal cells, dendritic cells, and innate-like T-cell populations. In particular, recent spatial transcriptomic work has highlighted layer-specific γδT- and MAIT-cell-associated niches in psoriatic inflammation (15, 16). In Crohn’s disease, spatial transcriptomic studies indicate that postoperative inflammation, stricturing disease, and fibrotic remodeling are organized within local epithelial, immune, stromal, and fibrotic niches rather than being diffusely distributed across the intestine (17, 18). Therefore, the convergence between psoriasis and Crohn’s disease lies in the use of related inflammatory modules, whereas the divergence lies in how these modules are spatially organized by skin versus intestinal tissue architecture.

These spatial data suggest that the shared TNF–IL-23–type 17 program is not deployed in the same tissue geometry. In psoriatic skin, inflammatory modules are arranged around epidermal–dermal keratinocyte–stromal–innate-like T-cell niches, whereas in Crohn’s disease they are embedded in epithelial injury zones, immune–stromal interactions, postoperative inflammatory fields, and fibrosis-associated niches. Thus, spatial transcriptomics supports convergence at the level of inflammatory modules, but divergence at the level of niche architecture, barrier repair, and tissue remodeling.

Taken together, the evidence linking psoriasis and Crohn’s disease is strongest for non-random association and pathway-level immune convergence, but weaker for direct causality or a unified disease identity. Epidemiological studies support bidirectional association, but remain vulnerable to confounding by disease severity, treatment exposure, surveillance bias, and shared comorbidities. Genetic and transcriptomic studies support convergence at the level of immune programs, especially cytokine signaling and type 17 immunity, but they do not explain tissue-specific outcomes. Spatial transcriptomics further shows that related inflammatory programs are organized in distinct tissue niches: epidermal–dermal keratinocyte–immune niches in psoriasis and epithelial injury, stromal remodeling, postoperative inflammation, or fibrosis-associated niches in Crohn’s disease. Therefore, the current evidence supports partial immune convergence shaped by tissue context, not a single shared disease process.

The major evidence domains supporting tissue-context-dependent immune convergence are summarized in **Table 1**.

**Table 1:** Evidence supporting tissue-context-dependent immune convergence between psoriasis and Crohn’s disease.

Evidence domain	Representative evidence	Key findings	Interpretive value	Main limitations	Key references
Epidemiological bidirectional association	Population-based studies and matched cohort studies	Patients with psoriasis have an increased risk of Crohn’s disease, especially when cutaneous disease is moderate to severe; patients with Crohn’s disease also have a higher risk of new-onset psoriasis than non-IBD controls.	Supports a non-random association between psoriasis and Crohn’s disease beyond coincidental comorbidity.	Observational data cannot establish causal direction and may be influenced by diagnostic definitions, treatment exposure, disease severity, and population structure.	(5, 7)
Disease severity and cross-organ inflammatory burden	Clinical cohorts, comorbidity/subgroup analysis	More severe psoriasis is associated with higher Crohn’s disease risk; Crohn’s disease with concomitant cutaneous psoriasis may show extensive intestinal involvement, perianal disease, elevated inflammatory markers, metabolic abnormalities, and poorer infliximab response.	Suggests that coexisting disease may mark a higher systemic inflammatory burden and a more complex cross-organ phenotype in selected patients.	Studies specifically focused on the psoriasis–Crohn’s disease subgroup remain limited; larger prospective validation is needed.	(5, 8)
Extraintestinal and cutaneous involvement	Clinical evidence on systemic psoriasis and IBD-related cutaneous manifestations	Crohn’s disease can be associated with cutaneous manifestations, while psoriasis is increasingly understood as a systemic immune-mediated disease rather than a purely localized skin disorder.	Supports cross-organ clinical assessment rather than single-organ interpretation.	Cutaneous manifestations are heterogeneous and must be distinguished from classic psoriasis, treatment-induced psoriasiform reactions, and other inflammatory dermatoses.	(1, 6, 59)
Genetic predisposition	GWAS, cross-trait GWAS, pleiotropy analysis	Shared susceptibility signals are enriched in immune-regulatory pathways, including cytokine signaling, lymphocyte differentiation, and IL-23-related biology.	Supports pathway-level genetic convergence rather than a single shared genetic cause.	Shared genetic signals do not prove disease identity and cannot explain organ-specific phenotypes or treatment outcomes alone.	(9–12)
Transcriptomic inflammatory convergence	Transcriptomics, integrative bioinformatics, immune-cell infiltration analysis	Overlapping gene-expression and pathway-enrichment signals are observed, particularly in immune activation and IL-17/IL-23-related programs.	Supports programmatic immune convergence between psoriasis and Crohn’s disease.	Transcriptomic overlap cannot establish causality or explain why similar upstream programs produce different tissue outcomes	(13, 14)
Spatially resolved inflammatory niches	Spatial transcriptomics and spatially informed single-cell studies	Psoriatic inflammation is organized within epidermal–dermal immune niches, whereas Crohn’s disease inflammation is embedded in epithelial injury, immune–stromal interaction, postoperative inflammatory fields, and fibrosis-associated niches.	Moves the review beyond cytokine sharing by showing that related inflammatory modules are organized differently in skin and gut tissue microenvironments.	Current spatial evidence remains disease-specific and does not yet define a unified psoriasis–Crohn’s disease spatial phenotype.	(15–18)
Share upstream immune nodes	Mechanistic studies and therapeutic translation evidence	TNF-α, IL-23, and IL-23-centered type 17 immunity contribute to chronic inflammatory maintenance in both skin and gut.	Provides a mechanistic framework for shared inflammatory architecture and explains why some upstream targets may have dual-organ value.	Not all shared immune mediators are interchangeable therapeutic targets because downstream effects are organ-specific.	(19–21, 33, 37, 96, 100, 103)
Therapeutic convergence and organ-specific tension	Biologic efficacy, safety evidence, IL-17-related intestinal concerns, and paradoxical psoriasis	TNF inhibition, IL-12/23 blockade, and selective IL-23 inhibition may provide cross-organ compatibility, whereas IL-17 blockade and paradoxical psoriasis illustrate organ-specific therapeutic risks	Supports the central therapeutic argument that shared upstream immunity can create opportunities, but tissue-specific biology creates therapeutic tensions.	Evidence is affected by differences in indications, trial design, prior biologic exposure, and limited studies in patients with both diseases.	(25–28, 38, 39, 51–55, 98, 99, 103, 114–116)

## 3 Shared immune architecture underlying psoriasis and Crohn’s disease

The immunological overlap between psoriasis and Crohn’s disease is most evident at the level of upstream cytokine signaling rather than complete disease identity. TNF-α signaling and IL-23-centered type 17 immunity are the most clinically relevant shared programs because they connect innate immune activation, adaptive immune amplification, barrier inflammation, and therapeutic translation. Additional IL-12/IFN-related and myeloid pathways contribute to the broader inflammatory background, but their cross-organ therapeutic relevance is less direct.

### 3.1 TNF-α: a shared upstream inflammatory amplifier

TNF-α is a central upstream inflammatory amplifier in both psoriasis and Crohn’s disease, connecting myeloid activation, chemokine release, leukocyte recruitment, and persistence of local tissue inflammation (19–21). In psoriatic skin, TNF-α supports the inflammatory loop among dendritic cells, T cells, and keratinocytes, promoting keratinocyte activation, chemokine and antimicrobial peptide release, and epidermal inflammatory amplification (19, 21–23). In Crohn’s disease, TNF-α is more closely linked to mucosal inflammation, epithelial injury, myeloid activation, barrier dysfunction, and tissue damage (20, 24). Thus, TNF-α participates in chronic inflammation in both diseases, but its downstream execution is shaped by different tissue compartments.

The clinical relevance of TNF-α is strengthened by the efficacy of TNF inhibitors in both moderate-to-severe cutaneous psoriasis and Crohn’s disease (25–28). TNF inhibition therefore represents one of the clearest examples of a shared upstream pathway that can be translated into dual-organ therapeutic benefit across skin and gut. However, this success should not be generalized to all shared immune pathways. When concomitant psoriatic arthritis is present, joint-specific disease activity should be assessed separately rather than being treated as an extension of cutaneous psoriasis.

### 3.2 IL-23-centered type 17 immunity: the core shared program connecting psoriasis and Crohn’s disease

IL-23 is a key upstream organizer of type 17 immunity in both psoriasis and Crohn’s disease. Its main role is to sustain, expand, and stabilize pathogenic Th17 cells and other IL-23-responsive immune populations, thereby maintaining chronic inflammatory programs (12, 29). This explains why IL-23-related pathways repeatedly appear in genetic, transcriptomic, mechanistic, and therapeutic studies of both diseases. In cutaneous psoriasis, IL-23 inhibition has become a central therapeutic strategy, and in Crohn’s disease, IL-23 is also recognized as an important therapeutic target (30, 31).

The downstream consequences of IL-23-centered immunity differ between skin and gut. In psoriasis, IL-23-driven type 17 responses promote IL-17- and IL-22-mediated keratinocyte activation, chemokine production, antimicrobial peptide release, neutrophil recruitment, and epidermal hyperplasia (21, 30). In Crohn’s disease, related type 17 programs operate within a mucosal environment where epithelial defense, microbial control, tight-junction regulation, and tissue repair are also required (29, 32). Thus, the overlap lies mainly in the IL-23-driven upstream program, not in identical downstream pathology. The downstream network of IL-23 includes IL-17A, IL-17F, IL-22, GM-CSF, and other mediators (33). Their biological meaning is organ-dependent: in psoriatic skin, they mainly amplify keratinocyte-centered inflammation, whereas in the gut they also contribute to mucosal defense, epithelial integrity, and repair responses (34, 35). This distinction is central to therapeutic interpretation. IL-23 can be viewed as a shared upstream node, whereas direct IL-17 blockade is a more organ-sensitive intervention and should not be assumed to be equally suitable for patients with Crohn’s disease.

### 3.3 Additional upstream modules: IL-12/IFN and myeloid priming

IL-12/IFN-related signaling and myeloid-cell priming also contribute to the shared inflammatory background of psoriasis and Crohn’s disease (19, 20, 36, 37). Historically, both diseases were partly interpreted through a Th1-related framework centered on IL-12-induced IFN-γ production (36). Although the IL-23/type 17 model now provides a more direct explanation for chronic inflammatory maintenance and tissue amplification, IL-12 remains relevant because it shares the p40 subunit with IL-23 and reflects upstream coupling between myeloid activation and T-cell polarization (37).

Clinically, the success of targeting p40 or p19 supports the importance of the IL-12/23 family in immune-mediated inflammatory diseases (38, 39). However, IL-12/IFN-related modules are less suitable than TNF-α or IL-23 as the central model for psoriasis–Crohn’s disease cross-organ translation. They more clearly represent enhanced antigen presentation, myeloid-cell priming, and type 1 inflammatory polarization, whereas IL-23-centered type 17 immunity is more directly linked to chronic inflammation and barrier-tissue amplification (40). For this reason, IL-12/IFN signaling and myeloid priming are discussed here as secondary contributors rather than as the main explanatory axis.

### 3.4 Why shared immunity produces organ-specific disease outcomes

Although psoriasis and Crohn’s disease share upstream immune programs such as TNF-α signaling and IL-23-centered type 17 immunity, these pathways do not operate in biologically neutral environments. Instead, they are filtered by distinct barrier architectures, resident immune ecologies, trafficking programs, microbial environments, and repair demands in the skin and gut. This tissue-specific filtering explains why similar inflammatory inputs can lead to divergent pathological phenotypes and therapeutic responses.

Barrier architecture is the first reason for this divergence. The skin is composed of keratinized stratified epithelium, in which keratinocytes function not only as structural barrier cells but also as active immune amplifiers (41). In contrast, the gut relies on mucus layers, a single-layer epithelium, tight junctions, and a mucosal immune system that must maintain tolerance and defense under constant exposure to food antigens and high-density microbiota (42). Therefore, type 17 inflammation is more readily translated into keratinocyte-centered epidermal hyperplasia, chemokine release, antimicrobial peptide production, and neutrophil recruitment in the skin, whereas related signals in the gut must also interact with epithelial repair, microbial control, mucosal defense, and immune tolerance.

Resident immune cells provide a second explanation. In psoriasis, skin-resident T cells, dendritic cells, stromal cells, and keratinocytes can persist after clinical remission and contribute to local inflammatory memory, which helps explain recurrent flares at previously affected sites (43, 44). By contrast, gut-resident immune cells are shaped by mucosal antigens, microbial metabolites, epithelial signals, and local tolerogenic mechanisms (45). As a result, the same TNF-α or IL-23-related program may be amplified as epidermal inflammation and local recurrence in the skin, but become more closely linked to epithelial injury, chronic antigen exposure, and impaired mucosal repair in the gut.

Leukocyte trafficking and tissue retention further separate the two organs. The skin and gut use distinct leukocyte imprinting programs: α4β7 integrin, CCR9, and their ligands are more closely associated with gut homing, whereas skin inflammation depends on different migration and retention pathways (46). Thus, even when systemic immune activation is shared, the destination, persistence, and local interaction partners of inflammatory cells remain organ-dependent. Microbiota load and repair requirements further amplify this divergence. Crohn’s disease develops in a mucosal environment continuously exposed to dense commensal microbiota and microbial metabolites, where host–microbiota interactions are central to disease maintenance (47). Although psoriasis is also influenced by barrier- and microbiota-related signals, the skin does not face the same microbial density or repair demand as the intestinal mucosa.

These differences also explain why blocking the same pathway may not produce equivalent clinical effects in skin and gut. Blocking IL-17 can directly interrupt keratinocyte-driven inflammation in psoriasis, but may disturb mucosal defense, epithelial repair, and barrier maintenance in Crohn’s disease or inflammatory bowel disease risk (29, 48–55). Conversely, therapies that successfully suppress intestinal inflammation may occasionally rebalance systemic cytokine networks in ways that promote new inflammatory outputs in the skin. These phenomena will be further explored below as the IL-17 paradox, paradoxical psoriasis, and broader therapeutic trade-offs.

**Figure 2:**
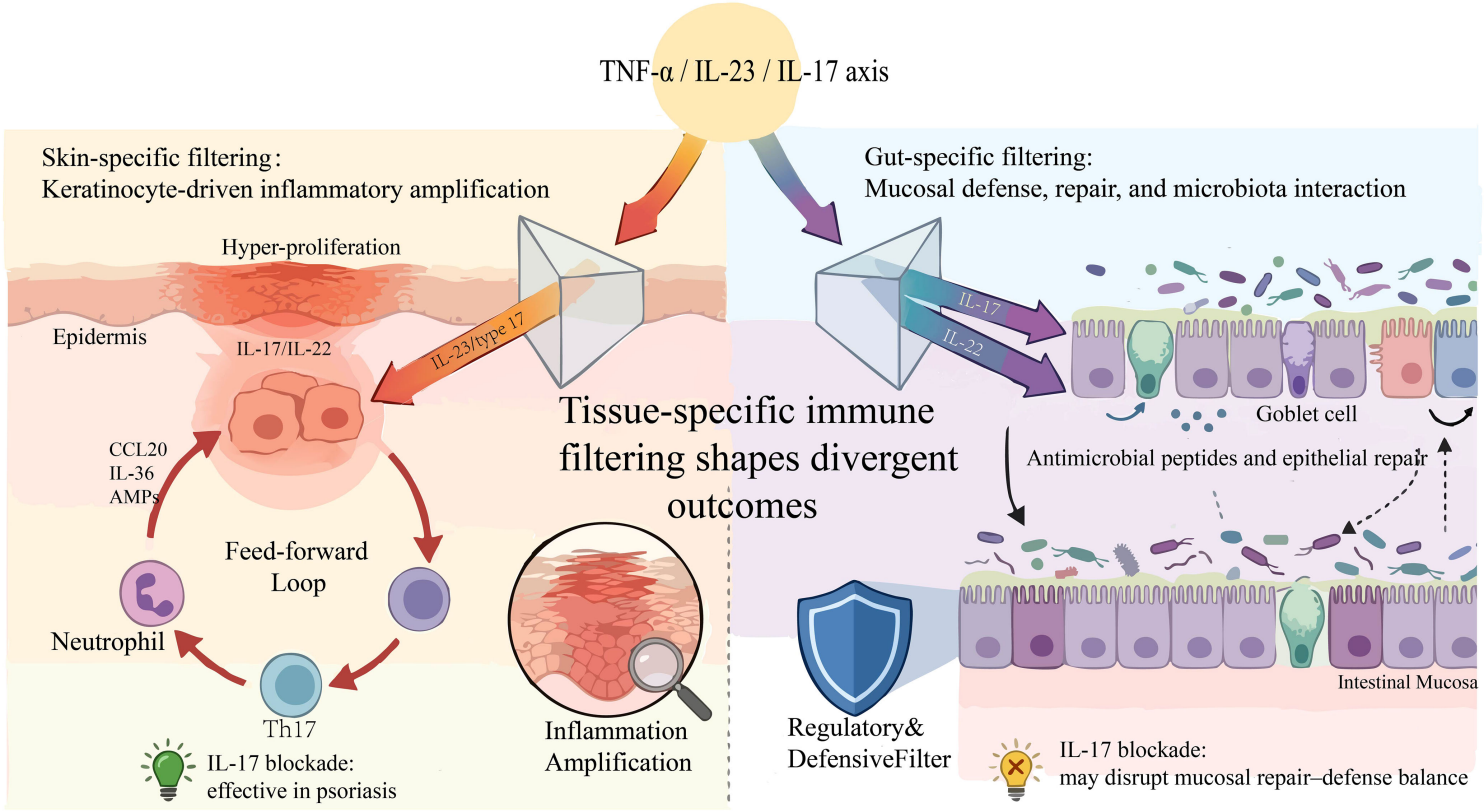
Tissue-specific interpretation of TNF-α/IL-23/type 17 inflammation in skin and gut. Although psoriasis and Crohn’s disease share TNF-α/IL-23/type 17-related signaling, these pathways operate within different tissue environments. In skin, type 17 responses amplify keratinocyte-centered inflammation, whereas in the gut they also intersect with mucosal defense, epithelial repair, and microbiota regulation. This distinction helps explain why IL-17 blockade is effective in psoriasis but may be unsuitable in patients with Crohn’s disease or inflammatory bowel disease risk. TNF-α, tumor necrosis factor α; IL, interleukin; Th17, T helper 17 cells; CCL20, C-C motif chemokine ligand 20; AMPs, antimicrobial peptides.

Taken together, shared upstream immunity does not mean that downstream mediators have identical roles in skin and gut. The shared immune architecture between psoriasis and Crohn’s disease should therefore be interpreted through organ-specific immune ecology before being translated into treatment decisions. This tissue-specific filtering model is summarized in **Figure 2**.

## 4 Gut-skin barrier and microbiota crosstalk

Cytokine overlap alone cannot explain why inflammation in two distant barrier organs may occur together. The gut–skin axis offers one possible explanation, but the evidence should be interpreted cautiously. Barrier dysfunction, microbial metabolites, immune-cell trafficking, and environmental exposures may connect intestinal and cutaneous inflammation, although direct human causal evidence remains incomplete.

### 4.1 The gut-skin axis: evidence hierarchy and causal limits

The gut–skin axis should be understood as a mechanistic explanation for cross-organ inflammatory communication rather than as a single proven causal route in humans. Potential links include circulating inflammatory cytokines, immune-cell trafficking, microbiota-derived metabolites, barrier dysfunction, and neuro–immune–endocrine signals (46, 56–59). Together, these mechanisms make cross-organ immune communication plausible, but they do not by themselves prove causality in patients.

Experimental evidence is stronger than current human causal evidence. In animal models, dermal injury has been shown to disrupt intestinal microbiome composition and intestinal immune homeostasis, supporting a skin-to-gut route of communication (60). Psoriasis-like dermatitis induced by imiquimod can also alter gut immunity, promote dysbiosis, and exacerbate colitis, while other models have linked psoriatic skin inflammation to intestinal inflammatory changes and systemic inflammatory responses (61–63). Conversely, gut dysbiosis and altered microbial metabolism can aggravate psoriasis-like skin inflammation, partly through enhanced Th17 infiltration and IL-23-related responses; short-chain fatty acid supplementation has shown protective effects in experimental psoriasis-like inflammation (64, 65). These findings indicate that gut–skin communication is biologically feasible and experimentally modifiable. However, the strength of evidence differs between experimental systems and human disease. In humans, current support for the gut–skin axis comes mainly from epidemiological association, cross-sectional microbiome studies, and indirect translational observations. Longitudinal studies that simultaneously profile skin lesions, intestinal inflammation, immune phenotypes, metabolites, and microbiota remain limited. Therefore, the gut–skin axis should be described as a potential amplifier or mediator of cross-organ inflammation, not as proof that psoriasis directly causes Crohn’s disease or that Crohn’s disease directly causes psoriasis.

### 4.2 Barrier dysfunction, microbiota, and metabolite-mediated immune tone

Barrier dysfunction provides a route through which local inflammation may become systemic immune exposure. In Crohn’s disease, disruption of mucus, epithelial integrity, tight junctions, and mucosal immune homeostasis increases contact between luminal antigens, microbial products, and mucosal immune cells, promoting mediators such as TNF-α, IL-1, IL-6, and IL-23 (42, 66–68). In psoriasis, epidermal inflammation and barrier-related abnormalities may contribute to systemic cytokine release and inflammatory spillover, supporting the view that psoriasis is not confined to visible skin lesions (1, 69, 70).

Microbiota and microbial metabolites further shape the direction and intensity of this inflammatory communication. In Crohn’s disease, dysbiosis is relatively consistent across studies and is commonly characterized by reduced microbial diversity, enrichment of pathobionts such as Enterobacteriaceae, and depletion of beneficial commensals such as Faecalibacterium (71). Pathologically relevant bacteria may also be enriched in the mucus layer or epithelial-adjacent regions, which makes host–microbiota interaction more directly connected to mucosal inflammation (47, 72). In psoriasis, gut microbiome findings are more heterogeneous across cohorts, but altered microbial composition, Firmicutes/Bacteroidetes imbalance, and reduced short-chain fatty acid production have been reported (73, 74). Skin microbiota may also influence local barrier integrity, lipid metabolism, antimicrobial defense, and inflammatory tone; lesional psoriasis has been associated with shifts in Cutibacterium, Staphylococcus, and Corynebacterium as well as altered metabolic and oxidative stress pathways (75–77).

The functional importance of microbiota is largely mediated through metabolites. Short-chain fatty acids can regulate epithelial barrier function and immune tolerance (57). Secondary bile acids participate in gut immune homeostasis and may promote regulatory T-cell differentiation while restraining excessive Th17 responses (78). Tryptophan-derived metabolites and aryl hydrocarbon receptor signaling provide another link between microbiota, mucosal barrier function, and immune regulation, particularly in inflammatory bowel disease (79). At present, these changes should be interpreted as modulators or amplifiers of inflammation rather than confirmed primary causes of psoriasis–Crohn’s disease overlap.

### 4.3 External modifiers and translational limits

Environmental and host-related modifiers may determine when the gut–skin axis becomes clinically relevant. Smoking, obesity, diet, antibiotic exposure, psychological stress, and infections can influence barrier integrity, microbiota composition, metabolic output, and systemic inflammatory tone (80). In Crohn’s disease, smoking is associated with increased disease risk and worse outcomes, possibly through effects on gut permeability, microcirculation, and cytokine responses (81). In psoriasis, obesity and metabolic inflammation can increase systemic inflammatory burden, promote gut microbiota imbalance, and reduce therapeutic response (82). Dietary patterns and antibiotic exposure may reshape gut microbial composition and metabolite production; in particular, antibiotic exposure is a well-established perturbator of the gut microbiome, although its specific relevance to psoriasis–Crohn’s disease overlap remains indirect (80, 83). Psychological stress may affect gut permeability and cytokine release through hypothalamic–pituitary–adrenal axis activation and neuroimmune signaling, while infections may act as inflammatory triggers in both psoriasis and Crohn’s disease (58, 80, 84, 85).

These external factors should not be presented as independent causes of psoriasis or Crohn’s disease. Their importance lies in their ability to modify the barrier–microbiota–immune network. In some patients, these modifiers may lower the threshold for cross-organ inflammatory amplification; in others, they may explain heterogeneity in disease activity, treatment response, or relapse pattern. This is especially relevant for patients with coexisting psoriasis and Crohn’s disease, because similar shared immune pathways may behave differently depending on smoking status, metabolic burden, diet pattern, antibiotic exposure, stress load, infection history, and previous biologic therapy.

Important translational limits remain. First, human causality has not been firmly established, and animal findings cannot be directly extrapolated into a confirmed bidirectional causal loop in patients. Second, the directionality of microbiome changes remains uncertain: dysbiosis may act as a driver, amplifier, consequence, or treatment-related effect depending on disease stage and therapeutic exposure. Third, methodological heterogeneity in sampling sites, sequencing platforms, reference databases, bioinformatic pipelines, and patient populations limits reproducibility across microbiome studies (86, 87). These limitations are particularly important because microbiome-based conclusions can vary according to whether fecal samples, mucosal biopsies, lesional skin, non-lesional skin, or treatment-naive populations are analyzed.

**Figure 3:**
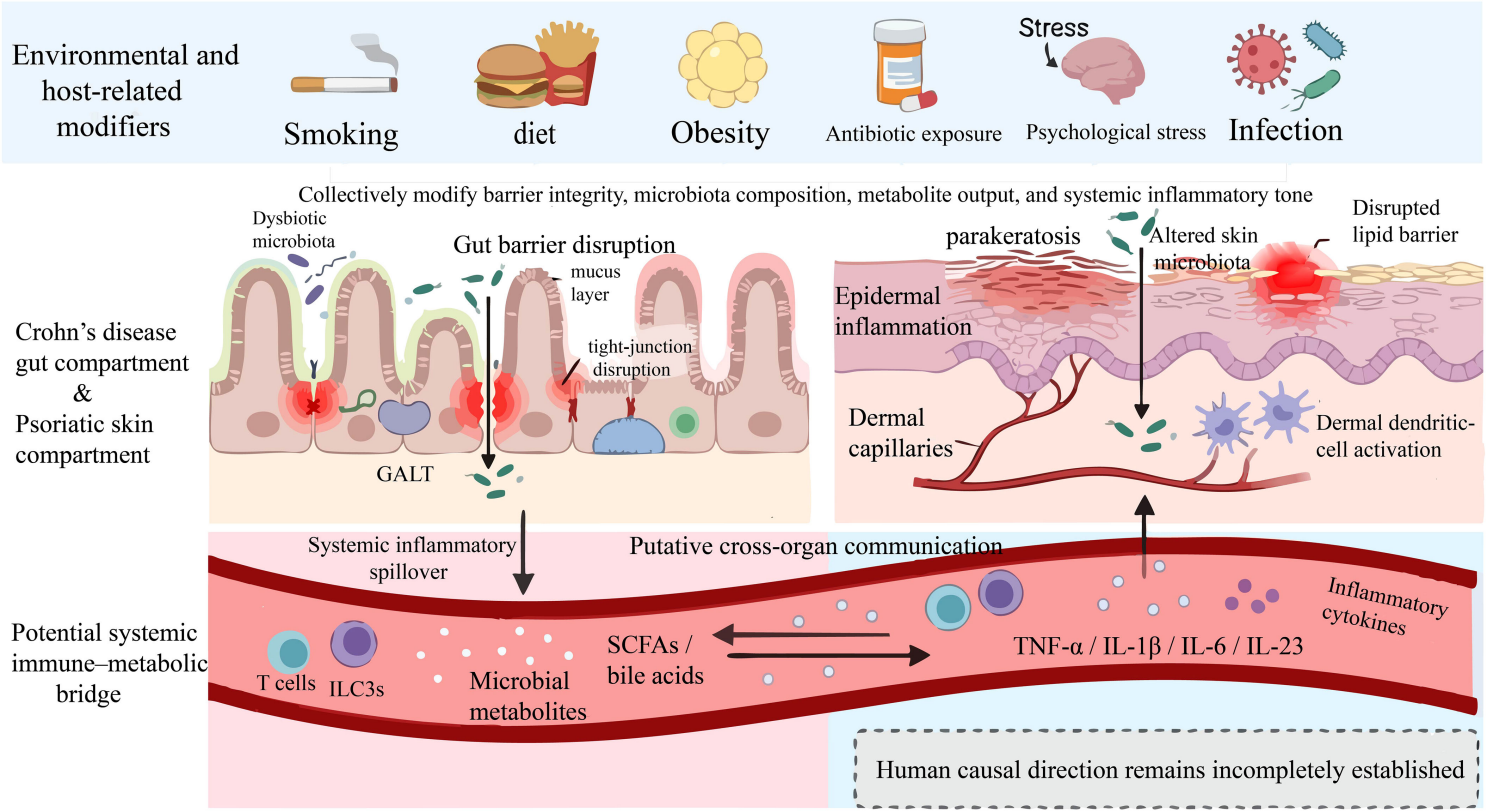
The gut–skin axis: barrier function, microbiota, metabolites, and external modifiers shaping cross-organ inflammation. The schematic shows how smoking, diet, obesity, antibiotic exposure, psychological stress, infection, and other host-related factors may influence gut and skin barrier integrity, microbiota composition, metabolite production, and systemic inflammation. In the Crohn’s disease gut compartment, barrier disruption may facilitate gut dysbiosis, microbial product exposure or translocation, GALT activation, and systemic inflammatory spillover. In the psoriatic skin compartment, epidermal inflammation and barrier-related abnormalities may contribute to altered skin microbiota, dermal dendritic-cell activation, and cytokine spillover. Circulating immune cells, inflammatory cytokines, and microbiota-derived metabolites, including SCFAs, bile acids, and tryptophan–AhR-related metabolites, are presented as potential bridges between gut and skin. Solid arrows indicate local barrier–microbiota–immune interactions, whereas dashed arrows indicate putative cross-organ communication. Human causal direction remains unresolved and requires stronger longitudinal and interventional validation. GALT, gut-associated lymphoid tissue; ILC3s, group 3 innate lymphoid cells; SCFAs, short-chain fatty acids; AhR, aryl hydrocarbon receptor; TNF-α, tumor necrosis factor α; IL, interleukin.

Overall, current evidence supports a possible gut–skin connection involving barrier dysfunction, dysbiosis, microbial metabolites, environmental exposures, and systemic inflammation. However, the direction of causality in humans remains unresolved, and microbiome findings remain limited by cohort differences and methodological heterogeneity. For psoriasis–Crohn’s disease overlap, the gut–skin axis should therefore be used to generate mechanistic hypotheses and guide patient stratification, rather than as proof that one disease directly causes the other or as a basis for routine microbiome-directed treatment. The proposed gut–skin axis and its major modifiers are summarized in **Figure 3**.

### 4.4 Traditional Chinese medicine perspectives on gut–skin inflammatory regulation

Traditional Chinese Medicine (TCM) provides a complementary clinical perspective for considering links between gastrointestinal symptoms, systemic imbalance, and skin manifestations. In this review, TCM concepts are discussed only as a traditional and clinical perspective; they should not be equated with the modern immunological gut–skin axis or with specific cytokine pathways such as the TNF-α/IL-23/IL-17 axis. Rather, TCM may provide a complementary conceptual model for exploring how gastrointestinal dysfunction, systemic inflammatory tone, and cutaneous inflammation are clinically connected, while modern mechanistic studies remain necessary to define the underlying cellular, molecular, and microbiota-related pathways. In the context of TCM, Crohn’s disease should not be regarded as the direct equivalent of a single classical disease entity but has been discussed in modern TCM research through syndrome-based interpretations related to chronic abdominal pain, diarrhea, dysentery-like conditions, intestinal ulceration, and recurrent inflammatory bowel symptoms.

Several clinical studies have investigated TCM-related interventions in Crohn’s disease and psoriasis, although the strength of evidence remains variable. For Crohn’s disease, recent systematic reviews and meta-analyses suggest that TCM may provide symptom relief in mild-to-moderate disease, but the included studies are heterogeneous and often limited by sample size, intervention variability, and differences in outcome assessment (88, 89). Acupuncture and moxibustion have also been evaluated in randomized studies of active Crohn’s disease, with reported effects on symptoms, intestinal microbiota, inflammation, and epithelial barrier-related markers, including tight junction protein expression (90–92). For psoriasis, oral Chinese herbal medicine and other TCM-based approaches have been assessed in systematic reviews and meta-analyses, with some evidence suggesting improvement in PASI or quality-of-life outcomes; however, methodological heterogeneity, variable formulations, limited high-quality trials, and insufficient long-term safety and recurrence data require cautious interpretation (93–95).

Therefore, TCM-related research should be positioned as a complementary and hypothesis-generating perspective for studying gut–skin inflammatory regulation, rather than as a substitute for guideline-based biologic or small-molecule therapy. Its relevance to this review lies mainly in potential intersections with barrier repair, microbiota modulation, microbial metabolite regulation, and systemic inflammatory control. Future studies should use standardized TCM interventions, rigorous randomized designs, objective inflammatory and barrier-related biomarkers, microbiome profiling, and long-term safety monitoring. Such work may help clarify whether selected traditional interventions can contribute to adjunctive management or mechanistic understanding in patients with psoriasis, Crohn’s disease, or overlapping gut–skin inflammatory phenotypes.

## 5 Therapeutic translation and organ-specific therapeutic tensions

For coexisting cutaneous psoriasis and Crohn’s disease, the therapeutic significance of shared inflammatory architecture lies not in whether a molecule is present in both diseases, but in whether targeting that molecule produces compatible effects across skin and gut. This distinction is essential because shared immune pathways may generate dual-organ therapeutic opportunities at upstream levels, while producing organ-specific risks at downstream or tissue-dependent levels. Therefore, therapeutic translation should be assessed according to three questions: which shared targets have demonstrated dual-organ value, which targets show organ-specific incompatibility, and how previous treatment exposure reshapes cross-organ decision-making.

### 5.1 Therapeutic translation: shared benefit and organ-specific tension

Drug response provides one of the most practical ways to judge whether a shared pathway is clinically meaningful. In coexisting cutaneous psoriasis and Crohn’s disease, the central issue is not whether TNF-α, IL-23, or type 17 immunity appears in both conditions, but whether intervention at a given node provides compatible benefit across both barrier organs. Current evidence supports TNF inhibition, IL-12/23 blockade, and selective IL-23 inhibition as the most plausible shared upstream strategies, whereas IL-17 blockade illustrates why downstream shared pathways may not be interchangeable therapeutic targets (25–28, 38, 39).

#### 5.1.1 Shared upstream targets with dual-organ value

TNF inhibitors have the most established clinical role across both cutaneous psoriasis and Crohn’s disease. Mechanistically, TNF-α is positioned upstream of myeloid activation, leukocyte recruitment, local tissue inflammation, and barrier damage (19, 20, 96). Clinically, TNF inhibitors remain an established systemic option for moderate-to-severe cutaneous psoriasis and a core biologic strategy for active Crohn’s disease, including induction, maintenance, and endoscopic outcomes (25–28). When cutaneous psoriasis, Crohn’s disease, and concomitant psoriatic arthritis are all clinically active, TNF inhibition may be particularly useful because it can cover intestinal inflammation, skin disease, and articular involvement. However, psoriatic arthritis should still be assessed as a separate disease domain rather than as an extension of cutaneous psoriasis (97).

Nevertheless, TNF inhibitors also illustrate the complexity of shared targets. Some patients experience primary non-response or secondary loss of response, and anti-TNF therapy is one of the most frequently reported settings for paradoxical psoriasiform reactions in inflammatory bowel disease (98, 99). Thus, TNF inhibition should be described as a mature dual-organ strategy, but not as a risk-free or universally compatible option.

IL-12/23 blockade provides another example of an upstream strategy with cross-disease relevance. By targeting the shared p40 subunit, this approach intervenes at a point linking myeloid activation, T-cell polarization, and chronic inflammatory maintenance (40, 100). Ustekinumab has established evidence in cutaneous psoriasis and Crohn’s disease, supporting the idea that selected upstream cytokine-family targets may provide clinically meaningful coverage across both skin and gut (101, 102). Nevertheless, p40 blockade affects both IL-12 and IL-23 pathways, and its relative position has changed as more selective IL-23 p19 inhibitors have become available.

Selective IL-23 inhibition may be even more closely aligned with the shared upstream type 17 program. The rationale is that IL-23 sustains pathogenic type 17 immunity while avoiding direct blockade of downstream IL-17-related functions that may be protective in the gut (12, 103). In cutaneous psoriasis, IL-23 inhibitors such as guselkumab, risankizumab, and tildrakizumab have shown strong and durable efficacy (104, 105). In Crohn’s disease, evidence for IL-23 p19 inhibition has expanded through risankizumab induction and maintenance studies, as well as more recent data on mirikizumab and guselkumab (39, 106–109). Therefore, selective IL-23 inhibition represents a highly plausible dual-organ strategy, especially for patients who require cutaneous disease control while maintaining intestinal compatibility.

The main limitation is that most evidence still comes from single-disease trials or mechanistic extrapolation rather than prospective studies specifically enrolling patients with coexisting psoriasis and Crohn’s disease. Thus, IL-23 inhibitors should be presented as a strong integrative direction, but not yet as a fully validated comorbidity-specific treatment algorithm.

Small molecules and emerging targeted therapies, including JAK, TYK2, and related intracellular signaling modulators, should be discussed cautiously (110, 111). Although these agents are relevant to cytokine-related intracellular signaling, their current indications, organ-specific efficacy, and safety profiles differ across cutaneous psoriasis, psoriatic arthritis, ulcerative colitis, and Crohn’s disease. Recent Crohn’s disease trials show that selected JAK inhibition can be clinically effective; for example, upadacitinib demonstrated efficacy as induction and maintenance therapy in patients with moderate-to-severe Crohn’s disease (112). However, this evidence should not be generalized to all intracellular signaling inhibitors. TYK2 inhibition has established relevance in plaque psoriasis, but recent randomized phase 2 studies of deucravacitinib in Crohn’s disease and ulcerative colitis indicate that TYK2-directed strategies remain insufficiently established for inflammatory bowel disease (113). Therefore, JAK/TYK2-related strategies should not be presented as an established skin-gut integration platform. At present, their role in coexisting cutaneous psoriasis and Crohn’s disease is best framed as individualized and context-dependent, particularly when established dual-organ strategies such as TNF, IL-12/23, or IL-23-directed therapies are unsuitable.

#### 5.1.2 Organ-specific risk: the IL-17 example

IL-17 is the clearest example of why shared immune biology does not automatically create an interchangeable therapeutic target. In cutaneous psoriasis, IL-17 is a highly effective therapeutic target because it directly interrupts keratinocyte-centered inflammatory amplification, including chemokine release, antimicrobial peptide production, neutrophil recruitment, and epidermal hyperproliferation (19, 21, 114). This explains the strong clinical value of IL-17 inhibitors in moderate-to-severe cutaneous psoriasis.

In Crohn’s disease, however, IL-17-related responses are embedded in mucosal defense, tight-junction regulation, antimicrobial protection, and epithelial repair (29, 34, 35). This tissue-specific role explains why IL-17 blockade has not translated into Crohn’s disease benefit and why clinical concern remains for new-onset or worsening inflammatory bowel disease during IL-17 inhibitor therapy. Previous IL-17A-targeting interventions in Crohn’s disease failed to show the same benefit seen in cutaneous psoriasis and were associated with worsening disease activity or adverse events in some studies (51, 52). More recent systematic reviews, pharmacovigilance analyses, and case summaries continue to report clinical signals of new or exacerbated inflammatory bowel disease under IL-17 inhibition, although the absolute risk appears low in the overall cutaneous psoriasis population (53–55).

Therefore, IL-17 should be framed as a shared inflammatory mediator but not as a stable shared therapeutic target for patients with Crohn’s disease. In patients with known Crohn’s disease, suspected intestinal inflammation, or high inflammatory bowel disease risk, IL-17 inhibitors should generally be avoided or used only after careful gastroenterological assessment.

#### 5.1.3 Paradoxical psoriasis and treatment-induced divergence

Paradoxical psoriasis represents a different type of therapeutic tension. Unlike the IL-17 example, where a downstream target is highly effective in skin but problematic in gut, paradoxical psoriasis occurs when an upstream therapy that benefits intestinal inflammation induces a new inflammatory phenotype in the skin. It is most often discussed in the context of anti-TNF therapy for inflammatory bowel disease and may present as new psoriasiform lesions or worsening of pre-existing cutaneous psoriasis despite improvement of intestinal inflammation (98, 99).

A systematic review and meta-analysis of inflammatory bowel disease patients receiving anti-TNF therapy reported psoriasis or psoriasiform lesions in approximately 6.0% of treated patients and identified factors such as female sex, younger age at treatment initiation, smoking, ileocolonic Crohn’s disease, and specific anti-TNF agents as associated risks (99). Mechanistically, paradoxical psoriasiform lesions may not be identical to classic idiopathic cutaneous psoriasis; they may reflect altered cytokine balance and innate or type I interferon-related inflammatory programs (115).

Paradoxical psoriasis therefore should not be interpreted as evidence that TNF is an ineffective shared target. Rather, it shows that successful control of one organ can rebalance immune networks in a way that produces a new tissue-specific inflammatory output in another organ. Rare psoriasiform reactions have also been reported with IL-12/23 or IL-23-targeted therapies, but anti-TNF therapy remains the most representative context for understanding this phenomenon (116).

### 5.2 Treatment selection in patients with coexisting cutaneous psoriasis and Crohn’s disease

When cutaneous psoriasis and Crohn’s disease coexist, treatment selection should not be based on choosing the strongest drug for each organ separately. Instead, decisions should be guided by four factors: the dominant organ burden, whether skin and intestinal activity are synchronized, previous biologic exposure and failure trajectory, and the cross-organ compatibility of the proposed mechanism (98, 99, 117–122).

If active Crohn’s disease is the dominant burden, especially with endoscopic activity, perianal disease, or systemic inflammation, intestinal control should usually be prioritized. In this setting, TNF inhibitors, ustekinumab, or selective IL-23 inhibitors may be considered according to prior exposure, contraindications, intestinal disease phenotype, and the severity of cutaneous psoriasis (27, 28, 39, 102, 106–109). If severe cutaneous psoriasis is dominant but Crohn’s disease is active or high-risk, IL-17 inhibitors should generally not be selected as the first-line systemic option because of intestinal safety concerns (51–55).

If cutaneous psoriasis coexists with concomitant psoriatic arthritis or other inflammatory joint manifestations (97), joint-specific efficacy should also be considered. TNF inhibitors may have practical value when Crohn’s disease activity, cutaneous psoriasis, and articular inflammation all require simultaneous coverage. However, psoriatic arthritis should be explicitly distinguished from cutaneous psoriasis, and joint involvement should not be assumed in all patients with psoriasis. Rheumatology assessment is needed when joint symptoms influence systemic treatment selection.

If new psoriasiform lesions appear or pre-existing cutaneous psoriasis worsens after anti-TNF therapy for Crohn’s disease, clinicians should first distinguish paradoxical psoriasiform reaction from uncontrolled pre-existing cutaneous psoriasis. Mild cutaneous disease may be managed with topical therapy while maintaining effective intestinal treatment. More severe, persistent, or recurrent lesions may require switching to another mechanism, such as ustekinumab or a selective IL-23 inhibitor, especially when intestinal disease control can be preserved (98, 99, 102, 106–109, 115).

Treatment selection should therefore begin with the patient’s dominant disease burden and prior treatment history, rather than with the name of a shared pathway. Shared upstream mechanisms support the use of TNF, IL-12/23, or IL-23-directed strategies in selected patients, but the final choice should depend on organ activity, biomarker profile, prior treatment response, safety risk, and whether skin and intestinal inflammation appear synchronized.

In clinical terms, shared upstream pathways create treatment opportunities, but organ-specific biology determines whether those opportunities are safe and useful for a given patient. TNF inhibition, IL-12/23 blockade, and selective IL-23 inhibition currently provide the strongest rationale for dual-organ therapeutic compatibility, whereas IL-17 blockade and paradoxical psoriasis demonstrate the limits of directly translating shared immune pathways into identical treatment effects. For patients with coexisting disease, the key clinical issue is whether the proposed mechanism matches the dominant organ burden, previous biologic exposure, and current inflammatory phenotype.

The main therapeutic opportunities, organ-specific tensions, and switching considerations are summarized in **Table 2**.

**Table 2:** Therapeutic opportunities and organ-specific risks in patients with coexisting cutaneous psoriasis and Crohn’s disease.

Therapeutic category or strategy	Main mechanism	Role in cutaneous psoriasis	Role in Crohn’s disease	Dual-organ compatibility	Main tensions or limitations	Key references
TNF inhibitors	Block TNF-α, an upstream inflammatory amplifier linking myeloid activation, leukocyte recruitment, barrier inflammation, and tissue damage.	Established systemic option for moderate-to-severe cutaneous psoriasis; also useful when psoriatic arthritis is clinically present and assessed separately.	Established biologic strategy for induction and maintenance of remission, especially in active or complicated Crohn’s disease.	High compatibility; one of the clearest examples of a shared upstream target with validated skin–gut benefit.	Primary non-response, secondary loss of response, and anti-TNF-associated paradoxical psoriasiform reactions may occur.	(19, 20, 25–28, 96–99)
IL-12/23 blockade	p40 blockade affecting the IL-12/23 cytokine family and upstream myeloid–T-cell polarization	Demonstrated efficacy in moderate-to-severe cutaneous psoriasis	Established evidence for induction and maintenance therapy in Crohn’s disease	Moderate to high compatibility; mechanistically coherent and generally skin–gut compatible.	Less selective than p19 IL-23 inhibition; response may vary by prior biologic exposure and disease phenotype.	(38, 40, 100–102)
Selective IL-23 inhibitors	Block p19 and target the IL-23-centered type 17 program without directly blocking IL-17.	Highly effective for moderate-to-severe cutaneous psoriasis, with strong and durable lesion clearance.	Increasing evidence in Crohn’s disease, especially for risankizumab, mirikizumab, and guselkumab.	High but still evolving compatibility; strong mechanistic rationale, but not yet a validated comorbidity-specific algorithm.	Most evidence comes from single-disease trials; prospective studies in patients with both diseases remain limited.	(12, 39, 103–109, 128)
IL-17 inhibitors	Block IL-17A, IL-17F, or IL-17 receptor signaling downstream of type 17 immunity.	Highly effective for cutaneous psoriasis by interrupting keratinocyte-centered inflammatory amplification	Not established as a stable Crohn’s disease therapy; IL-17-related responses may support mucosal defense and epithelial repair.	Low compatibility in patients with known Crohn’s disease or high inflammatory bowel disease risk.	Classic organ-specific therapeutic tension: effective in skin but may worsen or unmask intestinal inflammation.	(19, 21, 29, 34, 35, 51–55, 114)
JAK/TYK2 and related intracellular signaling modulators	Modulate JAK/STAT, TYK2, and related cytokine signaling pathways.	TYK2 inhibition is relevant to plaque psoriasis, but evidence is agent-specific.	JAK-related strategies are relevant to inflammatory bowel disease, but their Crohn’s disease role is drug-specific.	Context-dependent; mechanistically informative but not a stable dual-organ platform.	Indications and safety profiles differ across psoriasis, psoriatic arthritis, ulcerative colitis, and Crohn’s disease.	(110–113)
Switching strategy after anti-TNF-related psoriasiform lesions or organ-specific incompatibility	Switch from TNF blockade to a more compatible upstream mechanism, such as IL-12/23 or selective IL-23 inhibition.	May improve persistent, recurrent, or difficult-to-control psoriasiform lesions.	May preserve or restore intestinal control if the alternative mechanism has Crohn’s disease efficacy	A stratified management strategy rather than a single drug class.	Switching should not be automatic; clinicians must distinguish paradoxical psoriasis from uncontrolled pre-existing psoriasis and assess intestinal activity.	(98, 99, 102, 106–109, 115, 116)

### 5.3 Traditional and integrative therapeutic considerations

Traditional and integrative therapeutic approaches should be discussed separately from biologic and small-molecule therapies because their current evidentiary status and clinical role are different. In psoriasis, oral Chinese herbal medicine has been evaluated in systematic reviews and clinical studies, with some evidence suggesting improvement in Psoriasis Area and Severity Index (PASI) scores or quality-of-life outcomes, although the findings should be interpreted cautiously because of heterogeneity in formulations, trial design, and outcome assessment (93–95). Topical herbal preparations and traditional Chinese medicine bath therapy have also been studied as add-on approaches to conventional anti-psoriatic pharmacotherapy or phototherapy, but the evidence remains limited by methodological quality, intervention variability, and insufficient long-term safety and recurrence data (123, 124).

In Crohn’s disease, Chinese herbal medicine has been investigated mainly as an adjunctive approach, particularly in mild-to-moderate disease. Systematic reviews and meta-analyses suggest that TCM may improve selected clinical symptoms or disease activity indices, but the available evidence remains limited by sample size, intervention heterogeneity, and differences in outcome definitions (88, 89). Acupuncture and moxibustion have also been evaluated in randomized studies of active Crohn’s disease, with reported effects on clinical symptoms, intestinal microbiota, inflammatory responses, and epithelial barrier-related markers, including tight junction protein expression (90–92). Therefore, TCM-related interventions should be considered hypothesis-generating or adjunctive rather than core dual-organ therapeutic strategies for patients with coexisting psoriasis and Crohn’s disease.

From a clinical perspective, any use of traditional or integrative therapies in patients receiving corticosteroids, immunomodulators, biologics, or JAK/TYK2 inhibitors requires careful safety assessment. Potential concerns include herb–drug interactions, pharmacokinetic or pharmacodynamic interference, hepatotoxicity or nephrotoxicity, contamination or adulteration of herbal preparations, variability in active ingredients, and inadequate disclosure of herbal use by patients (125, 126). Future studies should therefore adopt standardized formulations or intervention protocols, rigorous randomized designs, predefined clinical and inflammatory outcomes, pharmacovigilance reporting, and long-term safety monitoring. These requirements are necessary before traditional or integrative interventions can be incorporated more confidently into cross-organ management frameworks for psoriasis and CD.

## 6 Patient stratification and integrated management in coexisting cutaneous psoriasis and Crohn’s disease

For patients with coexisting cutaneous psoriasis and Crohn’s disease, integrated management should begin with patient stratification rather than with a simple multidisciplinary referral model. The coexistence of the two diseases does not necessarily mean that every patient has the same shared inflammatory architecture. Some patients may represent a true cross-organ inflammatory phenotype, whereas others may have coincidental comorbidity, discordant disease activity, treatment-induced psoriasiform lesions, or organ-specific inflammatory programs. Therefore, the key clinical question is not only how to manage two diagnoses together, but how to determine whether cutaneous and intestinal inflammation are synchronized, discordant, treatment-induced, or dominated by one organ. This assessment should combine Crohn’s disease activity measures, cutaneous psoriasis severity, prior biologic exposure, and multidisciplinary dermatology–gastroenterology evaluation (98, 99, 119–122, 127).

Operationally, patient stratification should address four practical questions. First, are cutaneous and intestinal activities synchronized or discordant? Second, does the skin disease represent classic cutaneous psoriasis, worsening of pre-existing psoriasis, or a treatment-induced psoriasiform reaction? Third, is psoriatic arthritis present as a separate articular disease domain that may alter systemic treatment selection? Fourth, do available biomarkers, tissue profiles, or clinical phenotypes suggest TNF-dominant inflammation, IL-23/type 17-dominant inflammation, epithelial-injury/fibrotic inflammation, or microbiome-related inflammatory activity? This framework links clinical observation with mechanism-based stratification and helps identify patients most likely to benefit from dual-organ therapeutic strategies.

### 6.1 Defining the dominant organ burden

In patients with coexisting cutaneous psoriasis and Crohn’s disease, treatment decisions should begin by identifying the dominant organ burden. Some patients present with active intestinal inflammation as the primary driver of morbidity, whereas others are dominated by severe cutaneous psoriasis, special-site psoriasis, nail disease, treatment-induced psoriasiform reactions, or concomitant psoriatic arthritis. The dominant organ cannot be determined by symptoms alone. Assessment should integrate objective cutaneous severity and skin-related quality of life, endoscopic or radiologic intestinal activity, inflammatory biomarkers, perianal disease, nutritional status, comorbidities, joint symptoms when present, and prior treatment response (119–122, 127).

For Crohn’s disease, objective inflammatory assessment is particularly important because symptoms alone may not accurately reflect endoscopic or transmural activity. Serum CRP and fecal calprotectin can support monitoring and treatment adjustment, while discordance between symptoms and biomarkers may require endoscopic or radiologic reassessment before major therapeutic decisions are made (120). Therefore, intestinal activity should be evaluated not only by abdominal symptoms, but also by inflammatory biomarkers, endoscopic activity, radiologic disease behavior, perianal involvement, nutritional status, and prior treatment response.

For cutaneous psoriasis, stratification should include objective skin severity and patient-experienced disease burden. PASI, BSA, physician global assessment, DLQI, special-site involvement, and nail disease should be assessed because limited skin area can still produce substantial burden when lesions involve visible areas, scalp, genitals, palms, soles, or nails (122, 127). Concomitant psoriatic arthritis should be evaluated separately because joint involvement may change systemic treatment priorities even when the cutaneous burden is limited.

This distinction is clinically important because the dominant organ burden often determines treatment priority. If active Crohn’s disease is characterized by deep ulcers, persistent biomarker elevation, perianal involvement, stricturing or penetrating complications, or nutritional compromise, intestinal control should usually take priority while selecting a mechanism that remains compatible with cutaneous psoriasis and any concomitant psoriatic arthritis. Conversely, if intestinal inflammation is stable but the major burden comes from extensive plaques, special-site involvement, impaired quality of life, nail disease, or concomitant psoriatic arthritis, treatment should not simply follow a skin-first strategy; it should still avoid mechanisms that may worsen intestinal inflammation or create gut-specific risk (53, 117, 118, 127, 128).

Thus, dominant organ burden should be understood as a dynamic clinical state rather than a fixed label. A patient may shift from gut-dominant disease to skin-dominant disease after intestinal remission, or from skin-dominant disease to gut-dominant disease after relapse or treatment failure. Reassessment is particularly important after biologic switching, secondary loss of response, paradoxical psoriasis, new psoriatic arthritis, perianal complications, nutritional decline, or emerging metabolic and psychological burden (119, 129–133).

### 6.2 Identifying synchronized and discordant disease activity

Synchronized worsening of cutaneous psoriasis and Crohn’s disease may suggest a shared systemic inflammatory state or overlapping upstream immune activation. In such patients, TNF inhibition may be considered when a broad upstream inflammatory amplifier is clinically relevant in both organs (25–28), IL-12/23 blockade when p40-targeted therapy is appropriate across the skin–gut spectrum (101, 102), and selective IL-23 inhibition when IL-23-centered type 17 inflammation appears dominant while direct IL-17 blockade is undesirable because of intestinal risk (106–109, 128).

By contrast, discordant disease activity may indicate that the two organs are not currently driven by the same dominant mechanism. For example, active cutaneous psoriasis during intestinal remission may reflect a skin-dominant inflammatory state, inadequate cutaneous efficacy of the current therapy, or an independent cutaneous psoriasis trajectory. New-onset or worsening psoriasiform lesions during anti-TNF therapy should raise the possibility of paradoxical psoriasiform reaction rather than being automatically interpreted as worsening of classic cutaneous psoriasis.

This distinction also changes monitoring. For psoriasis patients, persistent diarrhea, recurrent abdominal pain, unexplained weight loss, anemia, elevated inflammatory markers, or perianal symptoms should prompt consideration of inflammatory bowel disease rather than being dismissed as nonspecific gastrointestinal discomfort (134, 135). For Crohn’s disease patients, persistent scaly plaques, scalp or nail involvement, palmoplantar lesions, genital lesions, or new psoriasiform eruptions during biologic therapy should prompt dermatological evaluation to distinguish classic psoriasis, paradoxical psoriasis, and other treatment-related skin reactions (98, 99).

Therefore, the purpose of bidirectional monitoring is not to test more indicators in every patient, but to determine whether disease activity across organs is synchronized, discordant, or treatment-induced. This classification directly affects therapeutic interpretation: synchronized activity may support a shared upstream inflammatory phenotype, whereas discordant activity should lead clinicians to reconsider dominant organ burden, treatment adequacy, paradoxical reactions, and organ-specific risk.

### 6.3 Biomarkers and tissue profiling for shared phenotypes

Not all patients with coexisting cutaneous psoriasis and Crohn’s disease share the same inflammatory architecture. Biomarkers and tissue profiling may help distinguish true cross-organ inflammatory phenotypes from coincidental comorbidity, discordant disease trajectories, or treatment-induced psoriasiform lesions. At present, stratification should begin with clinically validated measures. For Crohn’s disease, CRP, fecal calprotectin, endoscopic activity, radiologic disease behavior, perianal involvement, nutritional status, and prior treatment response help define objective intestinal inflammatory burden (120, 121). For cutaneous psoriasis, PASI, BSA, physician global assessment, DLQI, special-site involvement, nail disease, and prior treatment response remain central to clinical stratification (122, 127). Concomitant psoriatic arthritis should be assessed as a separate articular disease domain rather than as a severity extension of cutaneous psoriasis, because joint involvement may alter systemic treatment selection even when the cutaneous burden is limited.

Molecular biomarkers and multi-omics approaches, including transcriptomic, proteomic, microbiome, metabolomic, and spatial profiling, are increasingly being investigated in both psoriasis and inflammatory bowel disease. However, current evidence remains insufficient to support routine biomarker-guided systemic treatment selection in clinical practice. Their more realistic near-term value is to refine mechanism-based stratification by identifying whether TNF-α signaling, IL-23/type 17 immunity, myeloid activation, epithelial injury, stromal remodeling, fibrosis, or microbiome-related inflammation dominates in a given patient (136, 137).

Thus, biomarkers and tissue profiling should currently be viewed as complements to, rather than replacements for, clinical judgment. Future studies should define which clinical and molecular profiles identify patients most likely to benefit from dual-organ therapeutic strategies, rather than only comparing drug efficacy across broad diagnostic groups (119, 137, 138).

### 6.4 Integrated management based on stratification

Integrated management should be activated when patient stratification reveals cross-organ complexity, including synchronized skin and gut activity, discordant treatment responses, suspected paradoxical psoriasiform reactions, concomitant psoriatic arthritis or spondyloarthritis, perianal, fibrostenotic, or penetrating Crohn’s disease complications, prominent nutritional or metabolic burden, psychological distress, or diagnostic uncertainty (119–121, 130, 139, 140). Dermatology and gastroenterology should form the core of the care pathway, particularly when distinguishing active cutaneous psoriasis, paradoxical psoriasiform reactions, and Crohn’s disease activity. Input from rheumatology, clinical nutrition, radiology, pathology, endoscopy, or colorectal surgery may be required according to the dominant clinical problem, rather than applied uniformly to all patients.

Patient stratification is needed to determine whether shared inflammatory biology is clinically relevant in an individual patient. In coexisting cutaneous psoriasis and Crohn’s disease, management should not be organized simply around the presence of two diagnoses, nor should it rely on adding more specialty consultations. Instead, clinicians should determine the dominant organ burden, whether skin and intestinal activities are synchronized or discordant, whether prior therapies suggest mechanism-specific risk, and whether available biomarkers or tissue profiles support a shared inflammatory phenotype. In this way, integrated care becomes a mechanism-guided treatment strategy rather than a simple increase in specialist referrals (119, 137–139).

#### 7 Conclusions and future directions

In conclusion, psoriasis and Crohn’s disease are linked by partial immune convergence rather than by complete disease identity. Epidemiological, clinical, genetic, transcriptomic, spatial, and therapeutic evidence supports overlap at the level of selected upstream inflammatory programs, especially TNF-α signaling and IL-23-centered type 17 immunity. However, these shared programs are interpreted differently by skin and gut tissue ecologies, including barrier architecture, resident immune niches, microbial exposure, trafficking programs, and repair demands. This explains why similar upstream pathways can produce distinct tissue outcomes and divergent therapeutic responses.

Therapeutically, TNF inhibition, IL-12/23 blockade, and selective IL-23 inhibition currently provide the strongest rationale for dual-organ compatibility in selected patients, whereas IL-17 blockade and paradoxical psoriasiform reactions illustrate the limits of directly translating shared immune pathways into interchangeable treatment targets. Therefore, coexisting cutaneous psoriasis and Crohn’s disease should not automatically be managed as a single shared phenotype. Treatment decisions should be guided by dominant organ burden, synchronized or discordant disease activity, prior biologic exposure, psoriatic arthritis status, and objective inflammatory assessment.

Future studies should identify which patients with psoriasis and Crohn’s disease actually share a cross-organ inflammatory phenotype, rather than assuming that pathway overlap applies to all coexisting cases. This will require validated biomarkers, tissue-based profiling, longitudinal monitoring of skin and intestinal activity, and prospective studies specifically designed for patients with coexisting disease. This evidence is needed before shared immune biology can be used reliably to guide treatment in individual patients.
